# Dual role of CXCL10 in cancer progression: implications for immunotherapy and targeted treatment‎

**DOI:** 10.1080/15384047.2025.2538962

**Published:** 2025-08-04

**Authors:** Osama A. Madkhali, Sivakumar S. Moni, Yosif Almoshari, Fahad Y. Sabei, Awaji Y. Safhi

**Affiliations:** a Department of Pharmaceutics, College of Pharmacy, Jazan University, Jazan, Saudi ‎‎‎Arabia; b Health Research Centre, Jazan University, Jazan, Saudi Arabia

**Keywords:** CXCL10, therapeutic ‎strategies‎, tumor immune evasion ‎, signaling pathway, tumor microenvironment, immune regulation

## Abstract

CXCL10 is a chemokine crucial for immune cell recruitment and inflammation modulation, especially ‎within the tumor microenvironment.‎ This review critically analyzes the underexplored role of CXCL10 in modulating ‎JAK/STAT, MAPK/ERK, and PI3K/Akt pathways across different tumor types, highlighting ‎inconsistencies in current research and proposing novel therapeutic strategies based on ‎research ‎from databases such as PubMed and Scopus. Future targeted therapies could ‎include personalized ‎approaches that either enhance the immunostimulatory functions of CXCL10‎ or inhibit its tumor promoting effects. Techniques such as CRISPR/Cas9-mediated knockout of CXCL10 has demonstrated potential in preclinical models to ‎reduce tumor-promoting inflammation, while nanoparticle-based CXCL10 inhibitors enhance ‎immune checkpoint blockade efficacy in melanoma. In addition, targeting CXCL10-related mechanisms of immune evasion such as inhibition of CXCR3 may help to prevent metastasis. Futureresearch should focus on CXCL10-targeting approaches in highly immunosuppressive tumors, such as pancreatic and glioblastoma, where immune checkpoint inhibitors have shown limited efficacy.

## 1. Introduction‎

Cancer has long been considered a genetic disease caused by the accumulation of ‎mutations that ‎lead to uncontrolled cell proliferation. However, the role of the immune ‎system in shaping tumor ‎development and progression has become increasingly evident. ‎The immune system is responsible ‎for detecting and eliminating abnormal cells, including ‎those that have undergone malignant ‎transformation. This concept is known as cancer ‎immunosurveillance, a process in which the ‎immune system identifies and destroys ‎emerging tumor cells. When this process fails, tumors can ‎evade immune detection and ‎establish themselves in the host.^1,2^

CXCL10 is an interferon-gamma-inducible protein 10 (IP-10), which is a chemokine ‎that plays a crucial role in immune regulation and response. CXCL10 is primarily expressed by ‎macrophages and acts as an attractant for natural killer (NK) cells and T cells, contributing to the ‎regulation of T helper 1 (Th_1_) responses and IL-12-driven inflammation. This chemokine belongs to ‎the CXC chemokine family and is known for its ability to attract immune cells to sites of inflammation. Through its pro-inflammatory action, CXCL10 facilitates immune cell recruitment and ‎promotes cell infiltration, playing a central role in immune surveillance and inflammatory response ‎mechanisms.^3–5^ Initially CXCL10 discovered as an interferon-γ (IFN-‎γ)-inducible gene. ‎The CXCL10 has since emerged as a central player in both protective immunity ‎and pathological ‎immune responses, such as in autoimmune diseases, viral infections, ‎chronic inflammation, and ‎cancer.‎^6–9‎^ The interplay between the immune system and ‎cancer cells is highly dynamic, encompassing a spectrum from effective tumor elimination ‎to immune evasion.^10^ The immune ‎response in cancer is modulated by a complex network ‎of immune cells, signaling molecules which ‎collectively influence cancer outcomes. ‎Understanding the importance of the immune response in ‎cancer has led to the development ‎of novel immunotherapies, which harness the immune system’s ‎power to combat cancer more ‎effectively. Table 1 represents the cancer immune markers during innate ‎and adaptive phase ‎of cancer immunity. ‎

**Table 1.** Immune markers and their roles in cancer immunity.

Immune Markers	Descriptions		References
Natural killer (NK) Cell Receptors		NK cells play an important role in the immune response against cancer by recognizing and binding to stress-induced ligands such as MICA and MICB found on tumor cells. This recognition occurs mainly via the NKG2D receptor, which activates cytotoxic mechanisms that lead to the destruction of tumor cells. In contrast to T cells, NK cells do not need to encounter antigens earlier, which makes them crucial for the early detection of tumors. The binding of NKG2D to stress-induced ligands enhances the immune system’s ability to identify malignant cells and ultimately triggers apoptosis through the release of perforin and granzymes. However, tumors can evade the NK cell response by manipulating killer immunoglobulin-like receptors (KIR), which inhibit the cytotoxic actions of NK cells by downregulating activating ligands or altering the expression of MHC class I molecules. This evasion highlights the essential role of NK cell activity in preventing cancer progression and points to the potential of NK cells for immunotherapy.	^10^
Toll-Like Receptors		Toll-like receptors (TLRs), including TLR4 and TLR9, are key elements of the innate immune system found on immune cells such as dendritic cells (DCs) and macrophages. They recognize pathogen-associated molecular patterns (PAMPs) from microbes as well as damage-associated molecular patterns (DAMPs) released by stressed or dying cells. Upon activation, TLRs trigger signaling pathways that lead to the production of pro-inflammatory cytokines and promote the maturation of DCs, which enhances antigen presentation and stimulates the adaptive immune response. Inflammation triggered by TLRs can enhance anti-tumor immunity by facilitating the recruitment and activation of immune cells to combat cancer. However, excessive TLR activation can lead to chronic inflammation and create an environment that promotes tumor growth, angiogenesis and immune evasion. Given the dual role of TLRs in cancer, their modulation represents a promising therapeutic approach to either enhance the immune response or reduce tumor-supporting inflammation.	^11^
CD14+		CD14+ is a surface glycoprotein that is found on macrophages and monocytes and acts as a co-receptor for Toll-like receptors (TLRs), particularly TLR4. In cancer, CD14+ plays an important role in tumor-associated macrophages (TAMs), which create an immunosuppressive environment that promotes tumor growth and metastasis. Increased CD14+ expression in TAMs is typically associated with poor outcomes in solid tumors because it enhances the pro-tumorigenic activities of these cells, including increased inflammation, angiogenesis, and tissue remodeling. For this reason, CD14+ represents a promising target for cancer therapies aimed at altering the tumor microenvironment and preventing cancer progression.	^12^
CD68+	CD68+ is a glycoprotein that serves as an important marker for macrophages, especially those found in tissues. It is commonly used to identify cells of the macrophage lineage, including monocytes, histiocytes and tissue-resident macrophages. CD68+ macrophages are frequently observed in tumor-associated macrophages (TAMs) in the tumor microenvironment. These TAMs often play a role in promoting tumor growth, angiogenesis and immune evasion, making CD68+ an important marker for the study of tumor biology and the immune response to cancer.		^13^
CD163+	CD163+ is a scavenger receptor that is predominantly expressed on M2 macrophages, including tumor-associated macrophages (TAMs). It plays a key role in anti-inflammatory responses and tissue repair and contributes to the immunosuppressive environment in tumors. CD163+ TAMs are frequently associated with immunosuppression, tumor progression and metastasis in various cancers. High expression of CD163+ in the tumor microenvironment usually indicates a tumor-promoting role that helps cancer cells evade immune recognition and support tumor growth.		^14^
Fcγ receptors (FcγRs)	FcγRs are receptors that are expressed on immune cells such as macrophages, NK cells and neutrophils and bind to the Fc region of antibodies. This interaction is crucial for triggering antibody-dependent cellular cytotoxicity (ADCC), in which immune cells are activated to kill antibody-coated target cells, such as tumor cells. These receptors enhance the immune system’s ability to recognize and eliminate tumor cells, which contributes significantly to the efficacy of antibody-based cancer treatments.		^15^
Programmed Death-Ligand 1 (PD-L1)	PD-L1 is an immunoregulatory protein that is expressed on macrophages, dendritic cells, and various tumor cells. By binding to the PD-1 receptor on T cells, PD-L1 triggers T cell exhaustion, leading to reduced T cell activity and allowing tumors to evade immune recognition. This mechanism contributes significantly to immune escape in the tumor microenvironment, especially where tumor-associated macrophages (TAMs) have high levels of PD-L1. As a result, PD-L1 has become an important target for immune checkpoint inhibitor therapies, such as anti-PD-1 and anti-PD-L1 antibodies, which aim to restore T-cell function and enhance the immune system’s ability to combat cancer. These therapies have shown promise in the treatment of various cancers by blocking the PD-1/PD-L1 interaction and reactivating the immune response against tumors.		^16^
Myeloid-derived suppressor cells (MDSCs)	MDSCs are a heterogeneous population of immune cells characterized by markers such as CD11b and Gr1. They play a critical role in suppressing T cell activity and promoting immune evasion in the tumor microenvironment. MDSCs achieve this by secreting immunosuppressive cytokines such as IL-10 and TGF-β, which inhibit the immune response against tumors. Elevated levels of MDSCs are commonly observed in various cancers and are associated with poor prognosis as they contribute to immunosuppression and allow tumors to grow and metastasize by evading immune surveillance.		^17^
Siglec-9	It is an immunosuppressive receptor that is expressed on neutrophils and macrophages and modulates inflammation and immune tolerance by binding to sialic acids. In the tumor microenvironment, Siglec-9 promotes immune evasion and tumor growth by inhibiting macrophage and neutrophil functions, resulting in decreased anti-tumor immunity. This immunosuppressive function allows tumors to evade detection by the immune system, contributing to cancer progression and resistance to the immune response.		^18^
CD8+ cytotoxic T lymphocytes (CTL)	CTL are important immune cells responsible for the direct killing of tumor cells through mechanisms involving perforin, granzymes and FasL signaling pathways. These molecules trigger apoptosis in the target cells, which leads to the death of the tumor cells. A high infiltration of CD8+ T cells in the tumor microenvironment is usually associated with a better prognosis and better tumor control, as they improve the immune system’s ability to eliminate cancer.		^19^
CD4+ helper T	These are essential for the activation and regulation of other immune cells, such as CD8+ T cells and B cells. They play a critical role in generating effective anti-tumor responses by producing cytokines such as IFN-γ that improve immune function. CD4+ T cells help orchestrate the overall immune response by ensuring a coordinated attack on the tumor and contributing to sustained immune activation.		^20^
Granzyme B	It is a protease released by CD8+ T cells and NK cells that triggers apoptosis in target cells, including tumor cells. It is a critical mechanism by which cytotoxic T lymphocytes (CTLs) and NK cells eliminate cancer cells. Granzyme B enters the target cells and activates apoptotic signaling pathways, leading to cell death. Reduced granzyme B activity may indicate impaired anti-tumor immunity and weakens the body’s ability to combat tumors.		^21^
Perforin	It is a protein released by CD8+ T cells and NK cells that forms pores in the membranes of target cells through which granzymes can enter and trigger apoptosis. This mechanism is crucial for the immune system’s ability to eliminate tumor cells. Perforin deficiency impairs cytotoxic function, reducing the effectiveness of immune-mediated tumor elimination and potentially promoting cancer progression.		^22^
Interferon-γ (IFN-γ)	IFN-γ is a pro-inflammatory cytokine produced by CD8+ and CD4+ T cells and NK cells. It plays a critical role in activating macrophages, enhancing antigen presentation and promoting T cell responses, all of which are crucial for anti-tumor immunity. IFN-γ production is key to an effective immune response against cancer. However, many tumor cells develop resistance to IFN-γ signaling in order to evade immune recognition and ensure tumor survival.		^23^

CXCL10 is produced by a variety of cells in the tumor microenvironment, including ‎tumor cells, ‎stromal cells, endothelial cells, and infiltrating immune cells such as ‎macrophages and dendritic cells. ‎Its expression is primarily modulated by interferon-γ (IFN-‎γ) and tumor necrosis factor-α (TNF-α), ‎both of which are secreted during immune ‎responses. CXCL10 signals through its receptor CXCR3, ‎which is highly expressed on CD8+ ‎T cells, NK cells, and Th_1_-type CD4+ T cells.^24,25^ In tumors, ‎the expression of CXCL10 ‎is dynamic, often increasing in response to immune cell infiltration and ‎inflammation. This ‎chemokine plays a pivotal role in recruiting immune effector cells to the tumor, ‎initiating a ‎robust anti-tumor immune response. However, chronic inflammation and immune ‎‎dysregulation can lead to the persistent production of CXCL10, contributing to an ‎‎immunosuppressive microenvironment that favors tumor progression^10^. This review ‎aims to ‎provide an overview of the biological roles of CXCL10, its involvement in various ‎pathological ‎conditions, and its potential therapeutic applications in cancer. By analyzing ‎the current literature, ‎this article seeks to elucidate the mechanisms by which CXCL10 ‎functions, shedding light on its clinical ‎relevance and future research prospects.‎

## 2. CXCL10 signaling pathway

The structure of CXCL10 is critical for its efficient interaction with the CXCR3 receptor, which is ‎predominantly expressed on immune cells such as activated T cells, NK cells and ‎macrophages. CXCL10 has a high affinity for CXCR3, and this chemokine-receptor binding triggers ‎several intracellular signaling pathways that modulate immune cell trafficking and recruitment, activation and ‎function.^26^ The primary structure of CXCL10 consists of several crucial regions: the N-terminal ‎region, which is critical for receptor binding and initiation of signal transduction; the core domain, ‎which is responsible for stabilizing the protein through β-sheets and α-helices; and the C-terminal ‎tail, which ensures the structural integrity of the protein and its functionality in various biological ‎contexts. Together, these structural components enable CXCL10 to effectively mediate its immuno-‎modulatory and inflammatory functions. The major pathways are

### 2.1. JAK/STAT pathway‎

The Janus kinase (JAK)/signal transducer and activator of transcription (STAT) pathway is an ‎important signaling mechanism triggered by CXCL10 when binds to the CXCR3 receptor, it activates the receptor-associated JAK proteins, which then phosphorylate the STAT proteins. After ‎phosphorylation, the STAT proteins dimerize and migrate to the nucleus, where they modulate the ‎transcription of genes involved in immune cell activation, migration and cytokine production. The JAK/STAT signaling pathway plays a pivotal role in mediating the effects of CXCL10 signaling, particularly through the activation of JAK1, JAK2, and to some extent tyrosine kinase 2 (TYK2), which subsequently phosphorylate and activate STAT transcription factors. This signaling pathway promotes the expression of pro-inflammatory cytokines and chemokines, thereby driving the proliferation, differentiation and activation of various immune cells. In the context of the CXCL10–CXCR3 interaction, JAK/STAT signaling is essential for maintaining the functionality of immune cells, especially during infections and inflammatory responses. While this signaling pathway contributes significantly to protective immunity, its dysregulation e.g. chronic or excessive activation is associated with the development of autoimmune diseases, chronic inflammation and cancer. Activation of STAT1 is generally associated with antitumor immune responses that promote antigen presentation and cytotoxic T cell recruitment, while activation of STAT3 often promotes tumor cell survival, angiogenesis and immunosuppression. These contrasting roles highlight the context-dependent outcomes of JAK/STAT activation in CXCL10 signaling, which may be beneficial or detrimental depending on the tumor microenvironment, immune status, and co-activated pathways. Thus, the JAK/STAT signaling pathway serves as a key regulator at the interface between host defense and immune-mediated pathology.^27,28^

### 2.2. MAPK/ERK pathway ‎

The Mitogen-Activated Protein Kinase/Extracellular signal-regulated Kinase (MAPK/ERK) pathway is frequently activated in many cancers and plays a central role in ‎regulating cellular processes that control tumor growth, survival and metastasis. Abnormal activation of this pathway often results from mutations or overexpression of upstream growth factors, kinases or oncogenes such as Ras or BRAF. When CXCL10 binds to its receptor CXCR3 on ‎cancer cells or immune cells, it can activate the MAPK/ERK signaling pathway, leading to various ‎cancer-related processes. Activation of ERK in cancer cells promotes their survival and proliferation, while CXCL10-activated ERK in immune cells promotes the recruitment and activation of T ‎cells and NK cells, contributing to anti-tumor immunity.^29^ The MAPK/ERK pathway is also ‎crucial for modulating the tumor microenvironment by influencing the production of cytokines and ‎chemokines such as CXCL10, creating a feedback loop in which ERK activation promotes the secretion of chemokines, further activating immune cells.^30^ Therefore, CXCL10 plays a dual role in ‎cancer, in some cases, therapies targeting the MAPK/ERK signaling pathway can synergize with ‎immune-based therapies by increasing the expression of CXCL10 and recruiting immune effector ‎cells, which promotes immune activation. However, cancer cells can exploit the MAPK/ERK ‎pathway to modulate CXCL10 and the immune microenvironment in a manner that promotes ‎tumor evasion and metastasis, complicating therapeutic strategies to inhibit tumor growth.^31^

### 2.3. ‎PI3K/Akt pathway‎

The phosphoinositide 3-kinase/protein kinase B (PI3K/Akt) signaling pathway is a critical signaling mechanism in cancer that plays a central ‎role in the regulation of cell survival, growth, proliferation and metabolism.^32^ Dysregulation of ‎this pathway is common in various cancers and is a major contributor to tumor development, ‎progression and resistance to therapy. One of its main functions in cancer is to promote cell survival ‎by inhibiting apoptotic pathways. Akt, a key component of this pathway, phosphorylates and inactivates pro-apoptotic factors such as BAD and caspase 9, thus preventing programmed cell death.^33^ This anti-apoptotic effect enables cancer cells to evade death, even under stressful conditions ‎such as chemotherapy or radiotherapy, making them more resistant to treatment. The interaction ‎between CXCL10 and the PI3K/Akt signaling pathway plays a crucial role in the immune response, ‎cancer cell survival and overall cancer progression.^34^ When CXCL10 binds to its receptor CXCR3 ‎on immune or cancer cells, it activates multiple intracellular signaling pathways, including the ‎PI3K/Akt pathway. In cancer cells, this activation supports cell survival, promotes migration and ‎increases proliferation, all of which contributes to tumor growth and metastasis. In certain cancers, ‎CXCL10/CXCR3 signaling via the PI3K/Akt pathway helps cancer cells evade immune surveillance, allowing them to evade apoptosis and promote angiogenesis, which in turn promotes tumor ‎survival and spread. At the same time, CXCL10 can activate the PI3K/Akt signaling pathway in ‎immune cells such as T cells and NK cells to enhance their survival, proliferation and functionality.^35^ This increased immune cell activity boosts anti-tumor immunity by enabling these immune cells ‎to survive in the tumor microenvironment, even when confronted with stress signals and immunosuppressive factors in the tumor. Understanding the dual role of CXCL10 and the PI3K/Akt ‎signaling pathway in both cancer and immune cells provides valuable insights for the development ‎of therapeutic strategies aimed at either inhibiting cancer progression or enhancing the immune ‎response, thus highlighting potential opportunities for targeted cancer therapy.‎

### 2.4. Integrated pathway dynamics of CXCL10 signaling

**Figure 1.**
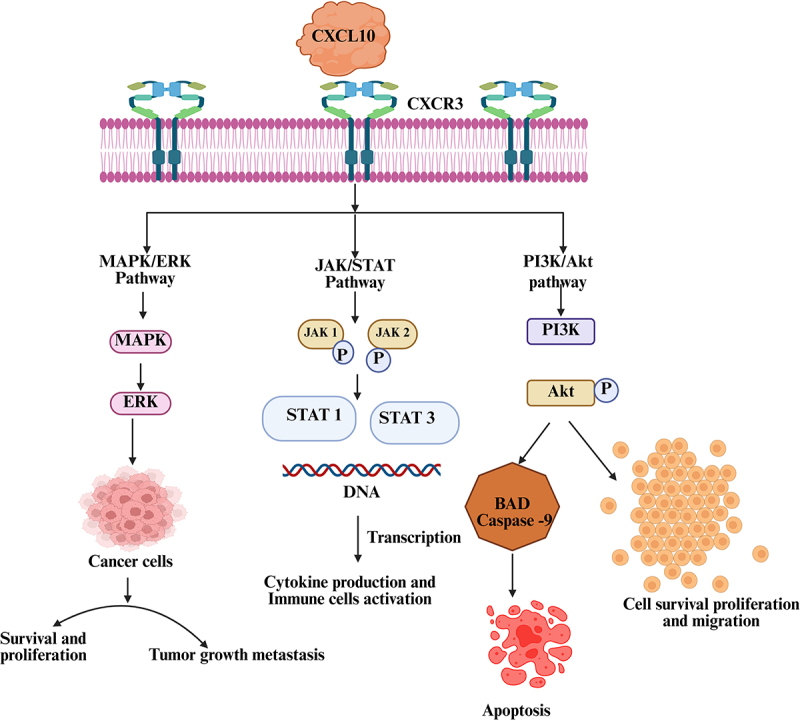
Mechanistic overview of CXCL10-mediated signaling pathways in cancer immunity.

The CXCL10–CXCR3 signaling axis activates several crucial intracellular signaling pathways JAK/STAT, MAPK/ERK, and PI3K/Akt that are interconnected (Figure 1) and together coordinate immune cell recruitment, inflammation, and cell behavior in the tumor microenvironment. The JAK/STAT signaling pathway primarily promotes the transcription of interferon-stimulated genes and inflammatory mediators, particularly through the activation of STAT1, thereby enhancing various immune cells, such as T cells, macrophages, and NK cells, to the tumor site.^31^ The MAPK/ERK signaling pathway, activated via CXCR3 in a G-protein-coupled receptor-dependent manner, supports cytokine production and promotes the migration of immune cells to inflamed or tumor-bearing tissues.^31^ In parallel, the PI3K/Akt signaling pathway contributes to cellular survival, proliferation, and metabolism by phosphorylating downstream effectors such as mammalian target of rapamycin (mTOR) and Bcl-2-associated death promoter (BAD), thereby supporting the persistence and function of recruited immune cells. These signaling pathways do not function independently but intersect at multiple nodes, and their interplay ensures a finely tuned immune response to both infectious and tumor-related stimuli. This integrated signaling contributes to shaping the tumor’s immune landscape and determines the strength and character of antitumor immunity.^36^

Despite this synergy, the functional outcomes of these pathways in response to CXCL10 are highly context-dependent and may differ between tumor types. For example, in melanoma, renal cell carcinoma, and other tumors with strong interferon signaling, CXCL10 enhances STAT1-driven T-cell activation. It supports ERK-mediated antiangiogenic effects, thereby promoting tumor regression and improving the response to immune checkpoint inhibitors.^37,38^

In contrast, in pancreatic, colon or glioblastoma tumors, CXCL10 can activate alternative signaling pathways such as STAT3, which promotes immunosuppression, or PI3K/Akt, which supports tumor cell proliferation and resistance to apoptosis. These variations result from differences in the expression of the receptor isoform (CXCR3-A and. CXCR3-B), the presence or absence of co-stimulatory cytokines, and the mutation status of the tumor.^26,39^ In some cases, chronic exposure to CXCL10 leads to T-cell exhaustion and decreased cytotoxic activity. In contrast, in other cancers, it favors immune exclusion and the formation of fibrotic barriers that prevent immune infiltration. Thus, while the same signaling pathways are activated downstream of CXCL10, their cellular targets and biological consequences differ substantially depending on tumorigenic and microenvironmental factors.^40,41^

The complexity of CXCL10-mediated signaling is further enhanced by its interaction with key oncogenic pathways, particularly the p53 and NF-κB pathways. In tumors with intact p53 function, CXCL10 signaling can enhance the DNA damage response and sensitize tumor cells to chemotherapy, promoting apoptosis and therapeutic efficacy. In contrast, in p53-deficient tumors, CXCL10 may assume a role in promoting cell survival and drug resistance, primarily through the activation of the PI3K/Akt pathway, which supports tumor progression.^42,43^ CXCL10 also exhibits a reciprocal relationship with NF-κB signaling, as it is not only induced by NF-κB activation but can also enhance and sustain NF-κB activity, creating a feedback loop that amplifies inflammatory signaling within the TME. This creates a positive feedback loop that perpetuates chronic inflammation and drives immunosuppressive responses seen in cancers such as hepatocellular carcinoma and gastric cancer.^44^ In addition, increased CXCL10 expression and activation of its downstream signaling components are increasingly associated with resistance to therapies, including immune checkpoint inhibitors and conventional chemotherapeutics.^45^

In resistant tumors, CXCL10 may facilitate the recruitment of Treg or MDSCs that dampen antitumor immunity and limit treatment success.^46^ Therefore, the CXCL10–CXCR3 axis acts as a dynamic modulator of the TME through its integration of JAK/STAT, MAPK/ERK, and PI3K/Akt signaling pathways, with STAT1 playing a central but context-dependent role that can mediate both protective and detrimental outcomes depending on tumor genotype, immune context, and cytokine milieu. Therefore, CXCL10 can either enhance anti-tumor immunity by recruiting cytotoxic T cells and activating inflammatory responses, or conversely contribute to immunosuppression and resistance to therapy, depending on the tumor context and the involvement of downstream signaling pathways. This duality represents both a challenge and an opportunity for targeted therapeutic interventions. Clinically, modulation of CXCL10 signaling offers a promising approach to reprogram the tumor microenvironment. For example, JAK/STAT inhibitors such as ruxolitinib and tofacitinib, currently approved for inflammatory and autoimmune diseases, are being investigated in cancer to suppress pathological immune activation and reduce chronic inflammation triggered by CXCL10, which could otherwise promote tumor growth and immune evasion. In tumors where overexpression of CXCL10 leads to STAT3 activation and immune suppression, JAK inhibitors may help redirect signaling toward a STAT1-dominant, pro-inflammatory response, which could improve the efficacy of immune checkpoint inhibitors. On the other hand, in cancers where CXCL10 supports anti-tumor immunity, care must be taken not to attenuate positive immune activity. Furthermore, targeting downstream effectors such as PI3K or Akt in combination with immunotherapies may counteract CXCL10-driven survival signaling in p53-deficient tumors, where the chemokine promotes resistance through the PI3K/Akt pathway. Similarly, modulation of MAPK/ERK signaling in conjunction with CXCL10 expression patterns could help reduce inflammation-driven tumor progression or support T-cell infiltration, depending on the immunological profile of the tumor. Overall, the integration of CXCL10 signaling profiles with pathway-specific inhibitors represents a rational therapeutic strategy that allows clinicians to tailor treatments based on the molecular landscape of the tumor and immune context.^46–48^

## 3. Tumor immune microenvironment and evasion

In the tumor immune microenvironment (TIME), CXCL10 plays a central role in the recruitment of key immune effector cells, including CD8^+^ cytotoxic T lymphocytes (CTLs), CD4^+^ Th1 cells, natural killer (NK) cells, macrophages and dendritic cells, primarily through its interaction with the CXCR3 receptor. These recruited cells contribute to antitumor immunity by promoting immune surveillance, facilitating antigen presentation and directly eliminating tumor cells. CXCL10also promotes a pro-inflammatory environment that enhances immune activation. However, tumors can develop strategies to evade this immune pressure by modulating CXCL10 gradients, suppressing the production of CXCL10, or inducing the accumulation of Tregs and MDSCs, both of which inhibit effector cell function. Prolonged exposure to CXCL10 within the TIME may also deplete T cells, reducing their cytotoxic potential. While CXCL10 plays an important role in coordinating effective immune responses against tumors, it can simultaneously be used by tumors to promote immune evasion and disease progression. CXCL10 also influences macrophage polarization and steers them toward an M1 phenotype that supports the antitumor response through the production of proinflammatory cytokines and reactive oxygen species. Conversely, CXCL10 can also attract tumor-associated macrophages (TAMs) with an M2 phenotype that promotes tumor growth through tissue remodeling, angiogenesis and immunosuppression.^47,49^ CXCL10 modulates the balance between immune activation and suppression by influencing the presence and function of Tregs and MDSCs. It promotes the infiltration of effector T cells and NK cells in tumors such as melanoma, renal cell carcinoma and non-small cell lung cancer, thus contributing to a better response to immunotherapy. However, in tumors with chronic inflammatory or immune-excluded phenotypes, such as pancreatic and colorectal cancers, CXCL10 can paradoxically promote the accumulation of Tregs and MDSCs. Tregs suppress immune activity by secreting anti-inflammatory cytokines such as IL-10 and TGF-β and inhibiting dendritic cell maturation, while MDSCs impair T cell activation via arginase-1 and ROS production. In such contexts, CXCL10 signaling may indirectly facilitate immune escape, particularly when enhanced by STAT3 activation or a hypoxic tumor microenvironment.^50,51^ Although CXCL10 is generally associated with the promotion of antitumor immunity, there is evidence that in certain tumor situations it can paradoxically promote metastasis through interactions with stromal components of the tumor microenvironment, such as fibroblasts, endothelial cells, and stromal macrophages. These stromal cells respond to CXCL10 signaling by producing factors that support tumor migration, invasion and vascular remodeling.^52^ ‎ A pivotal study by Wightman et al.^53^ showed that CXCL10 produced by stromal cells significantly contributes to metastasis in triple negative breast cancer (TNBC) by promoting motility and extravasation of CXCR3-expressing tumor cells to distant sites, particularly the lung. Depletion of CXCL10 in stromal cells reduced metastasis without affecting primary tumor growth, highlighting its stroma-specific, pro-metastatic role.^53^

Mechanistically, CXCL10 from the stroma activates CXCR3 on tumor cells and stimulates downstream signaling pathways such as MAPK/ERK and PI3K/Akt, leading to cytoskeletal reorganization, enhanced cell survival and targeted migration. These processes allow tumor cells to invade the extracellular matrix and enter the bloodstream. In addition, CXCL10 influences the composition of the immune system in metastatic niches by suppressing cytotoxic responses and promoting the polarization of M2 macrophages, which further facilitates tumor seeding. These findings underscore the dual function of CXCL10, which may act not as an immune activator but as a mediator of metastasis, particularly in the presence of CXCR3-expressing tumor cells and a reactive stroma. CXCL10 also plays a central role in enhancing Th1-like immune responses by attracting Th1 cells and CTLs, which are critical for effective tumor cell killing. It induces IFN-γ secretion, creating a positive feedback loop that enhances anti-cancer immune responses. In addition, CXCL10 exhibits angiostatic properties and helps limit tumor progression by suppressing the formation of new blood vessels, thereby reducing the supply of oxygen and nutrients to the tumor.^54^ Despite these protective effects, chronic inflammation induced by persistent expression of CXCL10 can lead to immune evasion, recruitment of suppressive immune cells and creation of a tumor-permissive microenvironment. CXCL10 also contributes to the modulation of immune checkpoints, particularly the PD-1/PD-L1 axis, which tumors exploit to inhibit immune function. The combination of CXCL10-targeted strategies with checkpoint inhibitors is emerging as a new approach to boost antitumor immunity and overcome immunosuppression.^55,56^ At the molecular level, CXCL10 is closely linked to the JAK-STAT signaling pathway. Its expression is usually triggered by proinflammatory cytokines such as IFN-γ, which activate the JAK-STAT cascade. When IFN-γ binds to its receptor, JAK1 and JAK2 are activated and subsequently phosphorylate STAT1. The phosphorylated STAT1 migrates to the nucleus and drives the transcription of immunoregulatory genes, including CXCL10. This upregulation enhances immune cell recruitment and supports immune surveillance by attracting CTLs, NK cells, macrophages and dendritic cells.^57,58^

The JAK-STAT pathway is critical for the generation of robust Th1 immune responses. CXCL10 enhances this pathway by maintaining the recruitment of Th1 cells and CTLs, further strengthening antitumor immunity.^59^ The positive feedback loop between IFN-γ and CXCL10 promotes sustained STAT1 activation and strengthens the overall anti-tumor cell inflammatory response. However, the consequences of sustained CXCL10 expression are highly context-dependent.^48^ In some cancers, chronic JAK-STAT activation and continuous CXCL10 expression can paradoxically facilitate tumor immune evasion. Persistent inflammation can lead to the recruitment of M2-like TAMs that support tumor survival through immunosuppression, tissue remodeling, and angiogenesis. In addition, tumors can use this pathway to upregulate immune checkpoint molecules such as PD-1 and PD-L1, thereby weakening the immune response. CXCL10 can modulate this process of immune suppression, highlighting its potential as a therapeutic target in combination with JAK-STAT inhibitors or checkpoint blockade therapies.^60,61^

CXCL10 is therefore an important player in cancer immunity, acting downstream of the JAK-STAT pathway to recruit immune cells, support Th1 responses and enhance immune surveillance. However, its chronic activation can shift its role toward tumor promotion by creating an immunosuppressive environment. Understanding the complex relationship between CXCL10, the JAK-STAT pathway and immune checkpoints provide valuable insights for the development of targeted therapies aimed at reprogramming the tumor microenvironment and improving cancer treatment outcomes.^62^

## 4. Therapeutic strategies on targeting CXCL10 in cancer‎ ‎

Therapeutic targeting of the CXCL10 and MAPK/ERK signaling pathways has gained ‎‎attention in cancer therapy as they play a central role in cancer progression, immune ‎modulation ‎and regulation of the tumor microenvironment. These signaling pathways ‎influence key processes ‎such as cell proliferation, survival, immune cell recruitment and ‎metastasis.^63^ Table 2 provides an ‎overview of targeted cancer therapies for CXCL10 and highlights strategies to enhance immune cell ‎recruitment, inhibit tumor ‎growth and reduce metastasis through specific interventions. ‎

**Table 2.** Anti-cancer therapeutic targeting of CXCL10‎.

Therapeutic Approach	Description	References
CXCL10 Agonists/Gene Therapy	CXCL10 agonists and gene therapy represent a promising approach to enhance immune cell recruitment in cancer by increasing CXCL10 levels in the tumor microenvironment, thereby boosting anti-tumor immunity. By attracting T cells, NK cells and macrophages to the tumor site. CXCL10 plays a crucial role in boosting the immune response against cancer cells. This strategy is particularly effective when combined with immune checkpoint inhibitors, such as anti-PD-1 and anti-CTLA-4 therapies, which help to unleash the full cytotoxic potential of the recruited immune cells. However, overexpression of CXCL10 also carries risks, as it can promote tumor growth, metastasis and immune evasion in certain cancers by fueling chronic inflammation or supporting the recruitment of suppressive immune cells such as tumor-associated macrophages (TAMs). Therefore, targeting CXCL10 requires precision to ensure that its expression is carefully orchestrated and localized in the tumor environment to maximize its immune-activating effect without inadvertently promoting cancer progression. Advances in gene delivery systems, targeted therapy and biomarker-guided patient selection will be crucial to optimizing the therapeutic potential of CXCL10 agonists and gene therapy in cancer.	^64–66^
CXCL10/CXCR3 axis inhibition	In CXCL10/CXCR3 axis inhibition, CXCR3 antagonists are used to block the binding of CXCL10 to its receptor, thereby disrupting the signaling pathways that promote tumor growth, immune evasion and metastasis. By inhibiting this interaction, the therapy aims to reduce cancer progression by limiting the tumor’s ability to use CXCL10 to suppress the immune system and spread metastases. The combination of CXCR3 antagonists with inhibitors of the PI3K/Akt pathway can enhance the therapeutic effect by promoting cancer cell apoptosis and limiting tumor survival. However, a major challenge is the potential reduction of the positive immune functions of CXCL10, such as the recruitment of T cells and NK cells, which are critical for anti-tumor immunity. Therefore, the immunomodulatory functions of CXCL10 must be carefully considered to ensure that tumor inhibition does not interfere with anti-cancer immune activation.	^67–69^
Inhibition of angiogenesis	Targeted inhibition of angiogenesis by CXCL10 is a promising approach to limit tumor growth and metastasis. CXCL10 plays a critical role in promoting the formation of blood vessels in certain tumors, and its blockade can effectively inhibit the tumor’s blood supply, cutting it off from essential nutrients and oxygen. This reduction in angiogenesis can lead to limited tumor growth and reduced metastatic potential. In addition, the combination of CXCL10 inhibitors with Vascular endothelial growth factor (VEGF) inhibitors or other angiogenesis-blocking agents could produce synergistic anti-tumor effects and enhance therapeutic success. However, the role of CXCL10 in angiogenesis could be tumor-specific, meaning that inhibition of CXCL10 could have different effects depending on the type of cancer, necessitating a tailored approach for different tumor situations.	^70,71^
CXCL10-neutralizing antibodies	CXCL10-neutralizing antibodies are being developed to prevent CXCL10 from binding to its receptor CXCR3 and thus effectively inhibit its tumor-promoting activities, particularly in metastasis and angiogenesis. By neutralizing CXCL10, these antibodies limit the tumor’s ability to spread and prevent the formation of new blood vessels that supply nutrients to the tumor, thereby suppressing tumor growth. This approach can be particularly effective when combined with angiogenesis inhibitors, allowing for better tumor control.	^3,26,72^

CXCL10, a chemokine, plays a ‎dual role in cancer by attracting immune cells such as T cells, NK ‎cells and macrophages to ‎the tumor site. Depending on the context, it can both promote anti-tumor ‎immunity and ‎contribute to tumor growth and metastasis. Increasing CXCL10 levels or enhancing signaling through CXCR3 receptors can boost the immune response by recruiting cytotoxic ‎T cells ‎and NK cells, which are critical for cancer cell clearance.^73^ This makes CXCL10 ‎a valuable target ‎in cancer immunotherapy, as therapies that enhance its expression could ‎increase anti-tumor ‎immune activity. In addition, CXCL10 has anti-angiogenic properties ‎by inhibiting the formation of ‎blood vessels and depriving tumors of important nutrients. In ‎cancers where CXCL10 inhibits angiogenesis and metastasis, targeting the CXCL10/CXCR3 ‎axis is a promising strategy to effectively ‎suppress tumor growth. However, the role of ‎CXCL10 in cancer is highly context-dependent, as its ‎effect can vary significantly ‎depending on tumor type and microenvironment.^74^ In some cases, ‎tumors manipulate ‎CXCL10 signaling to create an immunosuppressive microenvironment that ‎allows evasion ‎of the immune system and promotes metastasis. In these cases, CXCR3 antagonists ‎could be ‎used to block the tumor’s ability to use CXCL10 for immunosuppression, while in other ‎‎cases care must be taken to preserve the positive immune-activating effects of CXCL10. ‎Blocking the ‎CXCR3 receptor could reduce the recruitment of immunosuppressive cells or ‎attenuate the tumor-promoting effects of CXCL10, particularly in cancers where it ‎promotes metastasis and immune evasion.^75^ Therapies aimed at increasing CXCL10 expression in the tumor microenvironment have shown the potential to synergize with immune checkpoint inhibitors such as anti‑PD‑1 or anti‑CTLA‑4 therapies. By increasing CXCL10 levels, these strategies aim to improve the recruitment and activation of cytotoxic immune cells, thereby enhancing the overall immune response against the tumor.^76,77^ In melanoma patients, increased intra tumoral CXCL10 mRNA levels prior to treatment were found to be associated with favorable outcomes following PD‑1 blockade. This suggests that CXCL10 could serve not only as a therapeutic target but also as a predictive biomarker for the efficacy of checkpoint inhibitors. Further evidence for this concept is that intra tumoral vaccination with dendritic cells engineered to secrete CXCL9 and CXCL10 has been shown to enhance the T cell response and overcome resistance to PD‑1 therapy. Mechanistically, CXCL10 plays a key role by recruiting CXCR3^+^ CD8^+^ T cells to the tumor microenvironment, which in turn enhances IFN-γ production and sustains a Th1‑polarized immune response. In addition, CXCL10 contributes to vascular normalization, a process that improves the infiltration of immune cells into the tumor and further enhances the efficacy of checkpoint blockade therapies.

### 4.1. Gene therapy ‎

The main goal of CXCL10 ‎gene therapy is to restore or upregulate the expression of CXCL-10 ‎in ‎cancer cells or in the tumor microenvironment, thereby improving immune ‎surveillance and an‎ti tumor response while inhibiting tumor progression. By increasing ‎ CXCL10 expression, the strategy ‎aims to enhance immune responses and suppress tumor ‎angiogenesis.^49^ CXCL10 plays a critical ‎role in attracting immune cells ‎such as T cells, NK cells and macrophages to the ‎tumor, where they ‎recognize and destroy cancer cells, promoting tumor eradication. By facilitating ‎the ‎infiltration of immune cells into the tumor microenvironment, CXCL10 not only impairs ‎tumor ‎growth and metastasis, but also disrupts angiogenesis, which is essential for the ‎blood supply and ‎spread of the tumor. This dual action – improving immune cell function ‎while limiting angiogenesis ‎‎- underscores the significant potential of CXCL10 as a target in ‎cancer gene therapy. ^65,78 ‎^

CXCL10 gene therapy is a promising approach for the treatment of solid tumors such as ‎melanoma, ‎‎breast cancer, and lung cancer, where immune cell infiltration plays a crucial role ‎in disease control.^79,80^ Modern techniques such as CRISPR gene editing provide a precise ‎method to modulate gene ‎‎expression^81^ and can be developed to control the expression of ‎ CXCL10 in cancer cells, enabling ‎‎tailored therapeutic interventions. By targeting CXCL10 with ‎CRISPR, researchers can fine-tune ‎‎immune responses and specifically inhibit angiogenesis, ‎leading to a more controlled and tumor-‎specific therapeutic effect. In developing therapeutic ‎interventions, combining CXCL10 gene ‎therapy ‎with conventional cancer treatments such as ‎chemotherapy and radiotherapy could be ‎explored as ‎a synergistic approach to improve anti-‎tumor efficacy. In addition, developing nano ‎formulations ‎that deliver CXCL10 directly to target ‎sites offers a more precise method to ensure ‎ CXCL10 expression ‎specifically in cancer cells, ‎thereby increasing therapeutic benefit and specificity. ‎Personalized gene ‎therapy strategies that ‎adjust CXCL10 expression based on the molecular profile of ‎the individual ‎tumor offer the ‎potential for highly targeted and effective cancer treatments that ‎maximize the ‎immune ‎system’s ability to combat cancer. One of the significant challenges in CXCL10 ‎gene therapy is ‎to ‎control the immunostimulatory effect without causing excessive inflammation ‎that could lead ‎to ‎autoimmune reactions or tissue damage. In addition, the efficient and sustained delivery of ‎ CXCL10 to ‎tumor cells remains a technical hurdle, especially in heterogeneous tumors with ‎‎different immune ‎profiles. Future research will likely focus on optimizing delivery systems, ‎such as ‎using ‎nanoparticles or liposomes and refining CRISPR-based gene editing to increase ‎safety and ‎efficacy. ‎In the future, CXCL10 gene therapy could become a key component of ‎personalized cancer ‎treatment, ‎offering a novel approach to harness the immune system for ‎more effective cancer ‎therapies while ‎minimizing the risk of recurrence and metastasis. By ‎focusing on gene modulation, ‎personalized ‎strategies, and synergistic treatments, CXCL10 gene ‎therapy has the potential to ‎transform cancer ‎treatment. ‎

### 4.2. CXCL10 role in tumor immune evasion ‎

Cancer immune evasion presents a significant challenge for developing effective cancer ‎‎therapies. Despite considerable progress in understanding how cancer cells evade destruction by ‎the ‎immune system, strategies to combat these escape mechanisms are not yet fully developed. ‎Various ‎factors contribute to tumor persistence even when the host immune system functions. Table 3 ‎represents the mechanisms of CXCL10 in tumor immune evasion and progression and describes ‎‎how CXCL10 signals mainly through the CXCR3 receptor present on activated T cells and other ‎‎immune cells. ‎Under normal conditions, CXCL10 ‎promotes the recruitment of CTLs and T ‎helper cells into the TME, thereby ‎enhancing antitumor ‎immunity. ‎However, some tumors exploit CXCL10 to impair ‎immune function.^82^ By ‎overexpressing CXCL10, these tumors lead to prolonged ‎activation and subsequent desensitization ‎of CX ‎theCR3-expressing T cells, reducing ‎their ability to target and eliminate tumor cells and ‎leading to ‎immune exhaustion.^83^ ‎ In addition, tumors can manipulate CXCL10 to selectively attract Tregs that ‎suppress CTL activity and weaken the overall immune response, ‎thereby ‎promoting tumor survival. ‎CXCL10 also plays a role in the recruitment and ‎regulation of mye‎loid-derived suppressor cells ‎‎(MDSCs), potent antitumor immunity ‎inhibitors.^84^ In certain cancers, CXCL10 signaling ‎increases the accumulation of ‎MDSCs in the TME, where they release ‎immunosuppressive ‎substances such as ‎arginase-1, nitric oxide, and reactive oxygen species, further ‎inhibiting T cell ‎‎function. Through dysregulation of CXCL10 expression, tumors can shift the ‎‎immune milieu ‎toward immunosuppression, resulting in either the exclusion of T ‎cells from cancer ‎or the creation ‎of a locally immunosuppressive milieu.^85,86^ ‎During tumor growth, cancer cells ‎can undergo ‎mutations or epigenetic changes ‎that alter CXCL10 production, a process known as ‎‎immunoediting that allows ‎tumors to evade immune recognition. In addition, the CXCL10/CXCR3 ‎‎pathway ‎can promote evasion of the tumor immune system by stimulating angiogenesis. ^3 ‎‎ 87^ In ‎‎certain tumors, CXCL10/CXCR3 pathway promotes the formation of new ‎blood vessels that supply ‎‎the tumor with oxygen and nutrients while preventing ‎immune cells from invading the tumor core, ‎‎limiting an effective immune response.^88^

**Table 3.** Mechanisms of CXCL10 in tumor immune evasion and progression.

Perspective	Description	References
T-cell Recruitment & Function	CXCL 10 plays a critical role in the recruitment of CTLs and NKs to tumor sites, thereby enhancing antitumor immunity. However, tumors can exploit this mechanism by overexpressing CXCL10, leading to chronic T cell activation and subsequent exhaustion, which reduces the efficacy of the immune response. Furthermore, tumors manipulate this pathway to attract regulatory T cells (Tregs) that actively suppress the functions of CTL and NK cells, further weakening the immune system’s ability to attack the tumor. This immune evasion strategy is observed in cancers such as melanoma and breast cancer, where suppression of the immune system promotes tumor growth and survival.	^50,105,106^
T_reg_ Accumulation	CXCL10 supports immunosuppression by facilitating the recruitment of Tregs into the tumor microenvironment. The increased infiltration of Tregs leads to inhibition of cytotoxic T cell function, significantly dampening the antitumor immune response. This immune evasion mechanism allows tumors such as melanoma and hepatocellular carcinoma (HCC) to thrive by suppressing the body’s natural defenses and creating a microenvironment conducive to tumor growth and survival.	^82,107,108^
Myeloid-Derived Suppressor Cells (MDSCs)	CXCL10 facilitates the recruitment of MDSCs into the tumor microenvironment, where they play a critical role in suppressing immune responses. MDSCs inhibit the function of cytotoxic T cells by releasing immunosuppressive molecules such as arginase-1, nitric oxide and reactive oxygen species (ROS). These factors impair the activity of T cells and reduce the effectiveness of the immune system in combating and eliminating tumor cells. This mechanism is particularly pronounced in cancers such as breast cancer and hepatocellular carcinoma (HCC), where MDSCs contribute to the tumor’s ability to evade immune recognition and promote tumor progression.	^108^
Dysregulated CXCL10 Expression	Dysregulated CXCL10 expression can significantly influence the infiltration of immune cells in tumors and either promote or inhibit immune responses. Tumors often alter the expression of CXCL10 to reduce the recruitment of immune cells, particularly cytotoxic T cells, into the tumor microenvironment. By preventing the effective infiltration of immune cells, tumors create an immunosuppressive environment that promotes their survival and growth. This mechanism of immune evasion is observed in various cancers, including melanoma, breast cancer and hepatocellular carcinoma (HCC), where altered CXCL10 levels help the tumor evade immune surveillance and continue to grow unhindered.	^109–113^
Pro-Angiogenic Effects	The CXCL10/CXCR3 axis contributes to angiogenesis, i.e. the formation of new blood vessels that support tumor growth by supplying oxygen and nutrients. This pro-angiogenic effect plays a dual role by promoting tumor survival while hindering the infiltration of immune cells into the tumor microenvironment. By facilitating the development of a robust vascular network, tumors not only thrive but also create physical barriers that restrict the access of cytotoxic immune cells. This process is particularly important in cancers such as hepatocellular carcinoma (HCC) and melanoma, where angiogenesis driven by the CXCL10/CXCR3 axis promotes both tumor progression and evasion of the immune system.	^114–117^

### 4.3. CXCL10-targeting antibodies or small-molecule inhibitors

CXCL10-targeting antibodies are monoclonal antibodies specifically designed to bind and neutralize the chemokine CXCL10, preventing its interaction with the CXCR3 receptor. This blockade inhibits downstream signaling pathways such as JAK/STAT, MAPK/ERK and PI3K/Akt, which are normally activated by CXCL10-CXCR3 binding. These antibodies are gaining attention as novel therapeutic agents in cancers where chronic CXCL10 expression contributes to immune evasion, tumor progression and metastasis.^37,72^ In many tumors, elevated levels of CXCL10 lead to the recruitment of immunosuppressive cells such as regulatory Tregs, MDSCs and M2-polarized macrophages, which collectively dampen antitumor immunity. By neutralizing CXCL10, these antibodies can alter the tumor’s immunosuppressive microenvironment and potentially restore immune surveillance. In addition, CXCL10 antibodies are being explored in combination with immune checkpoint inhibitors such as anti-PD-1 or anti-CTLA-4 to improve therapeutic outcomes by enhancing effector T cell infiltration and reversing resistance to immunotherapy. While most CXCL10 antibodies are still in preclinical or early clinical development, they are also being investigated for inflammatory and autoimmune diseases such as rheumatoid arthritis, multiple sclerosis and inflammatory bowel disease, where aberrant CXCL10 expression plays a pathogenic role. Therefore, antibodies directed against CXCL10 represent a promising class of biologics that offer dual benefits by modulating chronic inflammation and reprogramming immune responses in cancer and immune-mediated diseases.^72,89^

### 4.4. Risks of targeting CXCL10 therapeutically in cancer

Targeting CXCL10 therapeutically poses significant risks due to its dual role in tumor immunity. While CXCL10 may contribute to tumor progression in certain contexts, particularly by recruiting immunosuppressive cells such as Tregs or M2-type macrophages, it is also a critical chemokine for orchestrating effective anti-tumor immune responses. CXCL10 facilitates the recruitment of CXCR3^+^ - CTLs, NK cells and Th1 cells into the tumor microenvironment, where they play an essential role in the recognition and destruction of tumor cells. Inhibition of CXCL10 can therefore impair immune cell trafficking, reduce IFN-γ-mediated immune activation and ultimately suppress anti-tumor immunity. This suppression could be particularly detrimental in cancers such as melanoma, non-small cell lung cancer (NSCLC) or renal cell carcinoma, where high intratumorally CXCL10 expression is associated with a better prognosis and increased response to immune checkpoint inhibitors such as anti-PD-1 and anti-CTLA-4 antibodies.^90,91^ In addition, CXCL10 has angiostatic properties, and its blockade may inadvertently promote angiogenesis, allowing tumors to grow and metastasize more efficiently. Permanent inhibition of CXCL10 could also disrupt chemokine gradients required for the trafficking of dendritic cells and effector T cells, further impairing immune surveillance. Consequently, therapeutic strategies targeting CXCL10 must be used with caution, as their use in immune-active tumor types may inadvertently lead to immunosuppression and compromise treatment efficacy. The context-dependent effect of CXCL10 underscores the need for personalized approaches and biomarker-guided patient selection when considering CXCL10-targeted interventions.

## 5. CXCL10 as a biomarker in diseases

CXCL10 serves as a reliable biomarker for immune system activation, inflammation and disease progression in various pathological conditions (Table 4). Its levels in biological fluids correlate with the severity of viral infections such as COVID-19 and hepatitis, making it a useful indicator of viral load and risk of cytokine storm. In autoimmune diseases such as rheumatoid arthritis, systemic lupus erythematosus and multiple sclerosis, elevated CXCL10 levels are a sign of an active immune response and disease exacerbation. It also serves as a predictive biomarker in cancer immunotherapy, indicating immune infiltration of the tumor and a potential therapeutic response. In transplantation medicine, CXCL10 is used to monitor acute rejection and graft-versus-host disease. Its consistent increase in various diseases underlines its utility as a noninvasive, sensitive and dynamic biomarker for diagnostic and prognostic applications.

**Table 4.** Diagnostic and prognostic utility of CXCL10 as a biomarker.

Disease/Condition	Biomarker Role	References
Viral infections	Elevated CXCL10 levels have been observed in viral infections such as COVID-19, hepatitis B and C, dengue and influenza, where its expression strongly correlates with viral load and disease severity. This makes CXCL10 a valuable biomarker for monitoring immune activation and predicting disease progression in viral diseases.	^91–93^
Autoimmune diseases	In autoimmune diseases such as rheumatoid arthritis, multiple sclerosis, systemic lupus erythematosus (SLE) and type 1 diabetes, CXCL10 levels are consistently elevated and reflect increased activity of the immune system. Its increased expression is associated with disease flares, tissue inflammation and immune cell infiltration, making CXCL10 a useful biomarker for assessing disease activity, monitoring disease progression and evaluating treatment response in autoimmune diseases.	^49,94,95^
Prognostic and Immunotherapy-Responsive Biomarker in cancer	Increased CXCL10 levels reflect the active infiltration of immune cells into the tumor microenvironment and are associated with an enhanced anti-tumor immune response. CXCL10 expression is associated with improved response to immunotherapy, particularly in tumors with a strong Th1-type immune signature. In addition, CXCL10 serves as a prognostic biomarker, whereby higher levels may correlate with better clinical outcomes in certain cancer types, highlighting its potential role in targeting therapeutic strategies and predicting treatment efficacy.	^96^
Bladder cancer (BLCA) prognosis and immune infiltration	A recent study has shown that CXCL10 is significantly upregulated in bladder cancer tissues and cell lines, which correlates with increased immune cell infiltration and favorable patient prognosis. High CXCL10 expression was associated with increased levels of immune and stromal components in the tumor microenvironment and accurately predicted response to immune checkpoint inhibitors.	^96^
Apoptosis mediation in esophageal cancer	In squamous cell carcinoma of the esophagus, activation of Toll-like receptor 3 (TLR3) by poly inosinic : polycytidylic acid (I:C) leads to strong production of CXCL10, which promotes tumor cell apoptosis and indicates a pro‑immunogenic tumor microenvironment.	^97^
Tumor-intrinsic IFN-α/CXCL-10 axis in improving immunotherapy	A study reported endogenous IFN-α was found to promote the expression of CXCL10 in tumor cells, which favors the recruitment and activation of CD8^+^ T cells. Tumors with high IFNα and CXCL-10 expression (“hot tumors”) responded better to anti‑PD‑L1 therapy, while low expressing “cold tumors” were resistant. Blocking CXCR-3 impeded T cell migration, highlighting the key role of the CXCL-10 and CXCR-3 axis in the efficacy of immunotherapy	^98^
Transplantation	CXCL10 has emerged as a valuable biomarker for the detection of immune-mediated complications such as allograft rejection and graft-versus-host disease (GVHD). Elevated levels of CXCL10 in serum, plasma or tissue samples are associated with increased immune activation and T-cell infiltration, which are characteristic of acute rejection episodes or the occurrence of GVHD. CXCL10 expression reflects interferon-γ induced inflammation within the transplanted organ or host tissue, making it a sensitive indicator for early diagnosis, disease monitoring and prediction of transplant outcome.	^99^
Pulmonary diseases	In pulmonary diseases such as chronic obstructive pulmonary disease (COPD), pulmonary fibrosis and asthma, elevated CXCL10 levels reflect underlying airway inflammation and immune cell recruitment. CXCL10 plays a key role in attracting activated T cells and other inflammatory cells to the lungs, contributing to tissue damage and disease progression. In COPD and pulmonary fibrosis, high CXCL10 expression correlates with disease severity and risk of exacerbation, while in asthma it is associated with Th1-dominant inflammatory responses. Thus, CXCL10 serves as a potential biomarker for assessing inflammatory activity, determining treatment strategies and monitoring disease progression in chronic airway diseases.	^100,101^
Sepsis/Systemic Inflammation	Elevated CXCL10 levels are closely associated with disease severity, immune dysregulation and increased mortality risk. As a chemokine induced by interferon-γ, CXCL10 contributes to the recruitment of immune cells such as T lymphocytes and Nk cells, thereby enhancing the inflammatory response. Its sustained elevation during systemic inflammation reflects a dysregulated state of the immune system and often marks the transition from protective immunity to harmful hyperinflammation. CXCL10 is thus a valuable biomarker for identifying patients at risk, predicting clinical outcomes and potentially guiding immunomodulatory interventions in sepsis and systemic inflammatory conditions.	^102,104^
Neuroinflammatory conditions	Neuro-HIV, encephalitis, multiple sclerosis (MS) and other neurodegenerative conditions with immune activation, CXCL10 is significantly elevated and plays a crucial role in mediating inflammation in the central nervous system (CNS). Its overexpression contributes to the recruitment of activated T cells and monocytes across the blood – brain barrier, exacerbating neuronal damage and disease progression. In multiple sclerosis, elevated levels of CXCL10 in CSF and serum correlate with disease activity and lesion formation. In neuroinfectious and viral encephalitis, CXCL10 also serves as a marker for the activation of the CNS immune system and has a prognostic value for monitoring the severity of the disease and the therapeutic response.	^103^

## 6. Conclusions

CXCL10 plays a multifaceted and context-dependent role in cancer progression, functioning both as a mediator of anti-tumor immunity and, paradoxically, as a promoter of immune evasion and metastasis. Its ability to recruit immune effector cells such as CD8^+^ -T cells, Th1 cells and NK cells via CXCR3 signaling makes it a valuable modulator of the tumor immunological microenvironment, particularly in cancers such as melanoma and renal cell carcinoma, where high CXCL10 expression correlates with a favorable response to immune checkpoint inhibitors and better prognosis. Current therapeutic strategies aim to exploit the immunostimulatory properties of CXCL10 through gene therapy, nanoparticle-based delivery or cytokine-induced upregulation to enhance immune cell infiltration, increase IFN-γ production and promote vascular normalization in the tumor microenvironment. As a possible approach is to use viral vectors such as lentiviruses or adenoviruses, as well as non-viral carriers such as nanoparticles and liposomes, may be employed to deliver the CXCL10 gene directly into tumor or immune cells to enhance local expression and boost the anti-tumor immune response. In malignant diseases characterized by persistent inflammation, fibrotic remodeling or immunosuppressive signals such as pancreatic cancer, glioblastoma multiforme and colorectal adenocarcinoma. Overexpression of CXCL10 may instead contribute to the recruitment and activation of immunosuppressive cell subsets, including Tregs, MDSCs and M2-polarized tumor-associated macrophages. This immunosuppressive shift can impair the function of cytotoxic lymphocytes, lead to T cell exhaustion and ultimately reduce the efficacy of immunotherapeutic measures. Resistance to therapy by activating downstream signaling pathways such as JAK/STAT3, PI3K/Akt and MAPK/ERK. In these contexts, CXCL10 promotes tumor survival, angiogenesis and metastasis, particularly in tumors with dysfunctional p53 or reactive stromal components. As a result, both CXCL10-enhancing and CXCL10-inhibiting therapeutic strategies are under active investigation, including monoclonal antibodies, CRISPR/Cas9-mediated gene modulation and small molecule inhibitors to modulate its specific activity.

Future research directions need to focus on the identification and validation of predictive biomarkers such as CXCL10 expression levels, CXCR3 receptor isoform distribution, dominance of STAT1/STAT3 signaling, and the presence of immunosuppressive stromal or immune cell populations. These parameters will be critical for clinical decision making and tailoring treatment to the individual immunogenomic profile of the tumor. Combinatorial therapy regimens combining CXCL10-targeted agents with ICIs, JAK/STAT inhibitors or PI3K/Akt pathway modulators should also be investigated in clinical trials, especially in resistant or immune-excluded tumor types. In addition, the potential risks of disrupting beneficial immune activation or inadvertently promoting angiogenesis when inhibiting CXCL10 in immune-responsive tumors must be carefully considered. This dual nature of CXCL10 emphasizes the importance of precision oncology in deciding when to suppress or stimulate its activity. Incorporating CXCL10 profiling into routine cancer diagnostics could not only help predict response to therapy but also prevent treatment failure due to inappropriate modulation. In the view of personalized treatment paradigms, CXCL10-targeted strategies promise to transform cancer immunotherapy by reprogramming the tumor microenvironment, overcoming resistance and achieving durable clinical responses.

## Data Availability

The data supporting the conclusions of this review article can be ‎found within the article itself.‎
